# Comprehensive analysis reveals a prognostic and therapeutic biomarker CD3D in the breast carcinoma microenvironment

**DOI:** 10.1042/BSR20202898

**Published:** 2021-01-07

**Authors:** Zhipeng Zhu, Weipeng Ye, Xiaofang Wu, Sihao Lin, Jiuhua Xu, Lulu Li, Jiayi Li, Haibin Wang, Zhengjie Huang

**Affiliations:** 1 Department of Gastrointestinal Surgery, Xiamen Cancer Center, The First Affiliated Hospital of Xiamen University, Xiamen, Fujian 361003, P.R. China; 2 Department of clinical medicine, Fujian Medical University, Fuzhou, Fujian 350004, P.R. China; 3 Department of Medical Oncology, Cancer Hospital, The First Affiliated Hospital of Xiamen University, Teaching Hospital of Fujian Medical University, Xiamen, Fujian 361003, P.R. China

**Keywords:** breast carcinoma, CD3D, prognostic biomarker, tumor microenvironment, tumor-infiltrating immune cells

## Abstract

Breast carcinoma (BRCA) is the most common carcinoma among women worldwide. Despite the great progress achieved in early detection and treatment, morbidity and mortality rates remain high. In the present study, we make a systematic analysis of BRCA using TCGA database by applying CIBERSORT and ESTIMATE computational methods, uncovered CD3D as a prognostic biomarker by intersection analysis of univariate COX and protein–protein interaction (PPI). It revealed that high CD3D expression was strongly associated with poor survival of BRCA, based on The Cancer Genome Atlas (TCGA) database and online websites. Gene Set Enrichment Analysis (GSEA) revealed that the high CD3D expression group was mainly enriched for the immune-related pathways and the low CD3D expression group was mainly enriched for metabolic-related activities. Based on CIBERSORT analysis, the difference test and correlation test suggested that CD3D had a strong correlation with T cells, particularly CD8 + T cells, which indicated that CD3D up-regulation may increase T cell immune infiltration in the TME and induce antitumor immunity by activating T lymphocytes. Furthermore, the correlation analysis showed that CD3D expression had a strongly positive correlation with immune checkpoints, which indicating that the underlying mechanism involves CD3D mediated regulation of T cell functions in BRCA, and single cell RNA-seq analysis revealed that CD3D correlate with CD8 + T cells and it is itself highly expressed in CD8 + T cells. In summary, we identified a prognostic biomarker CD3D in BRCA, which was associated with lymphocyte infiltration, immune checkpoints and could be developed for innovative therapeutics of BRCA.

## Introduction

Breast carcinoma (BRCA) is the most common carcinoma among women worldwide. In 2019, breast, lung and colorectal cancers were the top three cancers among women, accounting for 50% of the new diagnoses, while BRCA comprised 30%. The incidence of BRCA keeps increasing each year worldwide [1]. Despite great progress in early detection and treatment, morbidity and mortality remain high. Accordingly, there is urgent need for innovative therapeutics for BRCA. [2] Increasing evidence reveals that TME stimulates and promotes the growth and progression of tumors. The main components of TME include cancer cells and their supporting cells, such as resident stromal cells and infiltrating immune cells. Infiltration of immune cells, particularly T lymphocytes, have been reported to correlate survival in many types of cancers, including colon, ovarian and lung [3–6]. particularly contained CD4+ T-helper 1 (Th1) cells, CD4+ T-helper 2 cells (Th2) and CD8+ cytotoxic T cells (CTL). Th1 cells activate the antigen presenting cells, Th2 inhibit CTL function and promote tumor growth, and CTL cells act as tumor destruction immune cells. In BRCA, immune cells are mainly composed of T lymphocytes (70–80%), while the rest comprise B-lymphocytes macrophages, natural killer cells and antigen-presenting cells (APCs) (20–30%). Thus, TILs play a crucial role in tumor immunity.

Immunotherapy, including oncolytic viruses, vaccines, adoptive cell therapies, and most notably, immune checkpoint blockade (ICB), is currently the newest treatment modality for multiple types of cancers. However, immunotherapy in BRCA is still considered immunologically quiescent because of the low mutational burden compared with that for other cancers [7]. Several recent studies have indicated that TILs could be considered as a significant indicator of therapeutic response and survival prognosis [8–10]. As a vital portion of immunotherapy, ICB has revolutionized cancer therapy in multiple cancer types [11,12]. Nevertheless, no immune checkpoint antagonist has been approved for BRCA. Some small trials have obtained meaningful clinical results in patients with BRCA who were treated with CTLA-4 and PD-1/PD-L1 antagonists [2]. Meanwhile, several randomized phase 3 ICB monotherapy trials are ongoing. Thus, it is reasonable to develop novel candidates for immunotherapy of BRCA [7].

In the present study, the ESTIMATE and CIBERSORT computational methods were performed to assess immune-stromal scores and the ratio of immune and stromal components in BRCA samples. Kaplan–Meier survival analysis revealed that the immune component in TME can be used as a predictor of the prognosis of patients with BRCA. PPI-network, Kaplan–Meier survival analysis and Univariate COX regression analysis were applied and the predictive biomarker CD3D was identified. CD3D has been reported to correlate with multiply types of cancers, including colon cancer [13], bladder cancer [14], and glioblastoma [15]. CD3D participates in coding a protein complex, which is a vital portion of distinct chains, these chains could combine TCR and the ζ-chain to form the TCR–CD3 complex, which promotes T-cell activation [16]. However, few articles have focused on the association between CD3D and BRCA. Here, we performed a comprehensive analysis of CD3D in BRCA and the outcomes revealed a possible correlation between CD3D and tumor immune infiltration cells, immune checkpoints, and the mechanisms involved.

## Materials and methods

### Raw data acquisition

Gene expression profiles and clinical information were first downloaded from TCGA database (https://portal.gdc.cancer.gov/). Gene expression profiles contained 113 normal samples and 1109 BRCA samples. Survival data, including overall survival (OS), disease-free survival (DSS) and progression-free interval (PFI), were extracted from TCGA-Clinical Data

### Calculation of ImmuneScore, StromalScore and ESTIMATEScore

Estimate package was implemented to estimate the ratio of immune-stromal components in TME for each sample based on the ESTIMATE algorithm. Three kinds of scores were used to evaluate the ratio of immune and stromal components, and the sum of both, including ImmuneScore, StromalScore and ESTIMATEScore.

### Survival analysis between high-scores and low-scores of ImmuneScore, StromalScore and ESTIMATEScore

Based on the median scores of ImmuneScore, StromalScore, and ESTIMATEScore, Kaplan–Meier curves were generated using the ‘survival’ package. The log rank was used as the statistical significance test, and *P*<0.05 was considered as a significant cutoff criterion. The proportion of immune components was significantly associated with overall survival rate.

### DEGs were identified high scores and low scores based on ImmuneScore

The samples from BRCA patients were divided into high- and low- scores according to median scores of the ImmuneScore. Differentiation analysis was performed and differential expression genes (DEGs) were generated between the high- and low- score groups, while *P-*value<0.05 and absolute log fold change (FC)>2 were considered as the cutoff criterion. Package pheatmap was implemented to produce Heatmaps of DEGs. GO enrichment analysis based on DEGs was performed by using the packages enrichplot, clusterProfiler, and ggplot2, and terms with *P*<0.05 were considered to be significant.

### Identification of core genes

The protein–protein interaction (PPI) network of the DEGs was constructed by the Search Tool for the Retrieval of Interacting Genes (STRING; http://string.embl.de/) database, with a confidence of interactive relationship larger than 0.70. Cytoscape version 3.6.1 was used to reconstruct the PPI network, and the top ranked 30 genes were identified according to the number of nodes. Univariate COX regression analysis and Kaplan–Meier analysis for the DEGs were performed to identify genes significantly associated with survival prognosis (*P*-value<0.05). Intersection analysis was performed between the leading 30 nodes in the PPI network and the prognostic genes.

### Survival and independent analysis

Survival curves of differential CD3D expression were analyzed by mining the TCGA dataset, including OS, DSS and PFI. Furthermore, online websites were applied to draw survival curves, including GEPIA, Oncolnc and Kaplan–Meier Plotter. Univariate and multivariate COX proportional hazards analyses were performed to investigate the independence of CD3D in BRCA.

### Gene set enrichment analysis

GSEA was performed to investigate enriched KEGG pathways and groups of genes that may be associated with CD3D, all tumor samples was used for GSEA, 1000 repetitions of gene set permutations were performed. The nominal *P*-value and normalized enrichment score (NES) was utilized to selected significant pathways (nominal *P*-value<0.05).

### Tumor infiltrating immune cells associated with CD3D

The TIC abundance profile in BRCA samples was calculated by applying CIBERSORT method, with *P*<0.05 being considered for further study. The difference test of TICs between high and low CD3D was performed by applying the packages limma and vioplot. The correlation test between CD3D and 21 kinds of immune cells was conducted by applying the packages limma, ggplot2, ggpubr and ggExtra. The correlation between CD3D and the abundance of immune infiltrates in BRCA was performed by employing the TIMER (https://cistrome.shinyapps.io/timer/).

### Single cell RNA-seq data processing

Single cell RNA-seq data associated with breast cancer (GSE118389) was downloaded form GEO dataset. The Seurat package implemented in R was performed to identify major cell types. Highly variable genes were generated and used to perform PCA, t-SNE analysis was used to compute principle components.

## Result

### ImmuneScore was associated with survival in BRCA patients

**Figure 1:**
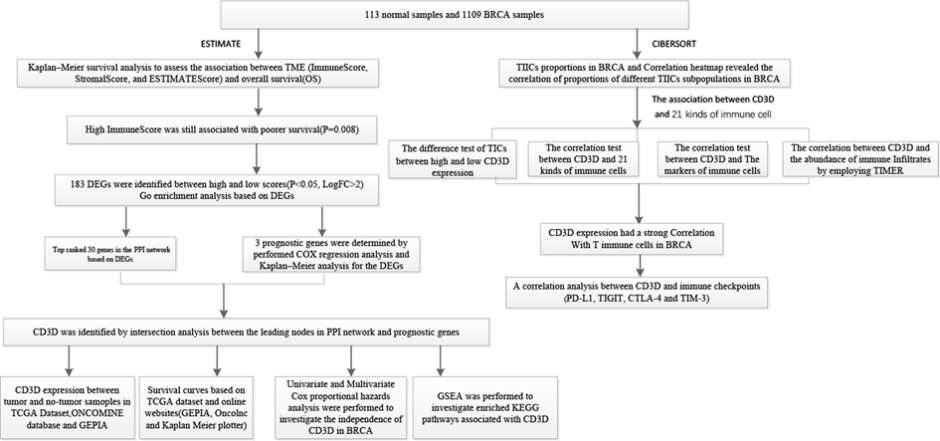
The schematic flow chart of the study

**Figure 2:**
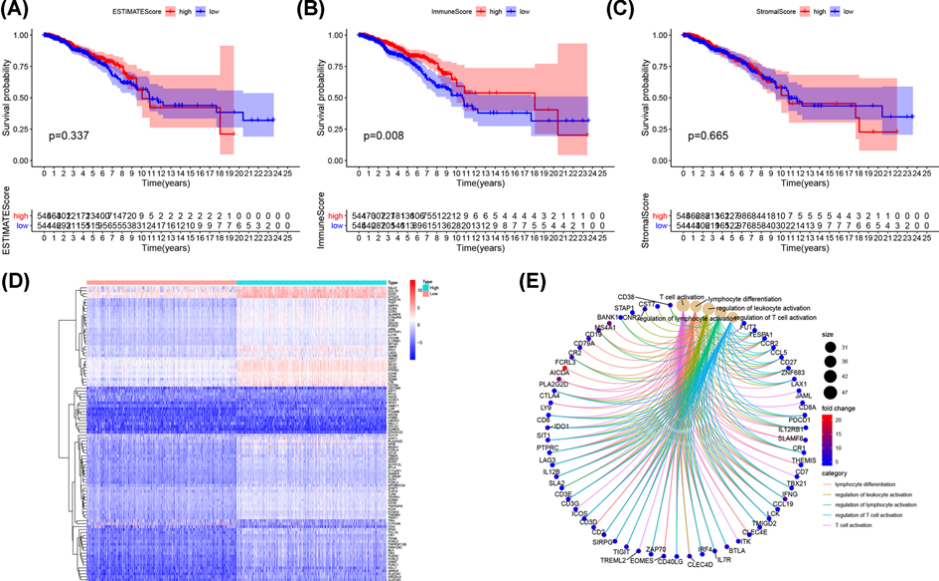
Kaplan–Meier survival analysis for scores and heatmaps and Go enrichment analysis for DEGs in immune components (**A**) Kaplan–Meier survival curve of ESTIMATEScore. (**B**) Kaplan–Meier survival curve of ImmuneScore. (**C**) Kaplan–Meier survival curve of StromalScore. (**D**) Heatmap for DEGs in immune components. (**E**) GO enrichment analysis for DEGs.

The design of our study is presented in Figure 1. We performed Kaplan–Meier survival analysis to assess the association between TME (ImmuneScore, StromalScore, and ESTIMATEScore) and OS. As showed in Figure 2, although there was no significant association between StromalScore (Figure 2C) or ESTIMATEScore (Figure 2A) and survival rate, we found that a higher ImmuneScore was associated with poorer survival (Figure 2B). The result indicated that the immune components in TME were able to predict the prognosis in BRCA patients.

### DEGs were identified according to ImmuneScore for Go enrichment analysis

To determine any possible changes in expression of genes involved in the immune component, analysis according to the median of lmuneScores (high score vs. low score) was used, and 13 up-regulated genes and 170 down-regulated genes were identified (Figure 2D). The DEGs may be the genes influencing the immune status. The gene ontology (GO) enrichment analysis indicated that the DEGs were associated with immune-related terms, including lymphocyte differentiation, regulation of leukocyte activation, regulation of lymphocyte activation, regulation of T cell activation, and T cell activation. The results indicated that the DEGs participated in immune-related activities and, therefore, immune components in TME may be considered as predominant features in BRCA patients (Figure 2E). We could also hypothesize that the up-regulated genes are protective factors that inhibit tumor development.

### Core genes correlated with pathomechanism and survival prognosis

**Figure 3:**
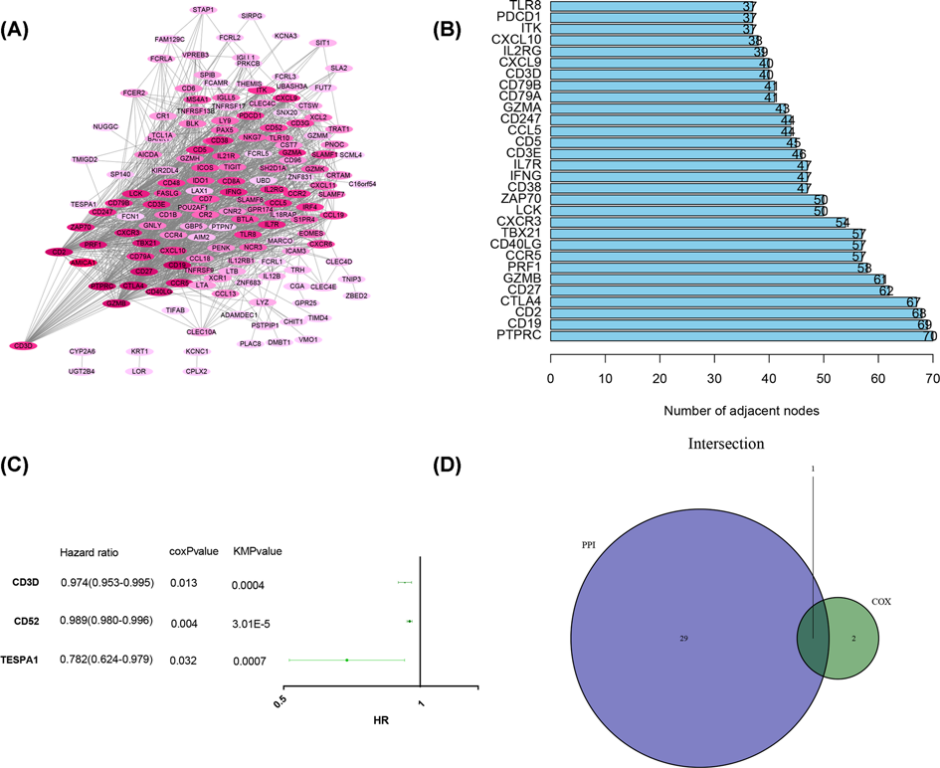
Core genes correlated with pathomechanism and survival prognosis was identified by performing protein–protein interaction network, Kaplan–Meier survival analysis, Univariate COX regression analysis and intersection analysis (**A**) Protein–protein interaction network with the nodes with confidence of interactive relationship larger than 0.70. (**B**) The top ranked 30 genes were ordered according to the number of nodes. (**C**) Kaplan–Meier survival analysis and Univariate COX regression analysis were performed to identify prognostic genes form DEGs. (**D**) Venn plot was draw to show the common genes between the top 30 nodes in PPI network and prognostic genes.

To further identify core genes associated both with the pathogenetic mechanism and survival prognosis, the PPI network was constructed based on the STRING database (Figure 3A), and the top 30 genes are demonstrated in Figure 3B. Kaplan–Meier survival analysis and Univariate COX regression analysis were performed to identify genes significantly associated with survival prognosis (Figure 3C). Next, intersection analysis between the top 30 genes in the PPI network was performed to identify genes significantly associated with prognosis, and only one core gene, CD3D, was identified (Figure 3D).

### Increased CD3D expression was associated with better prognosis

**Figure 4:**
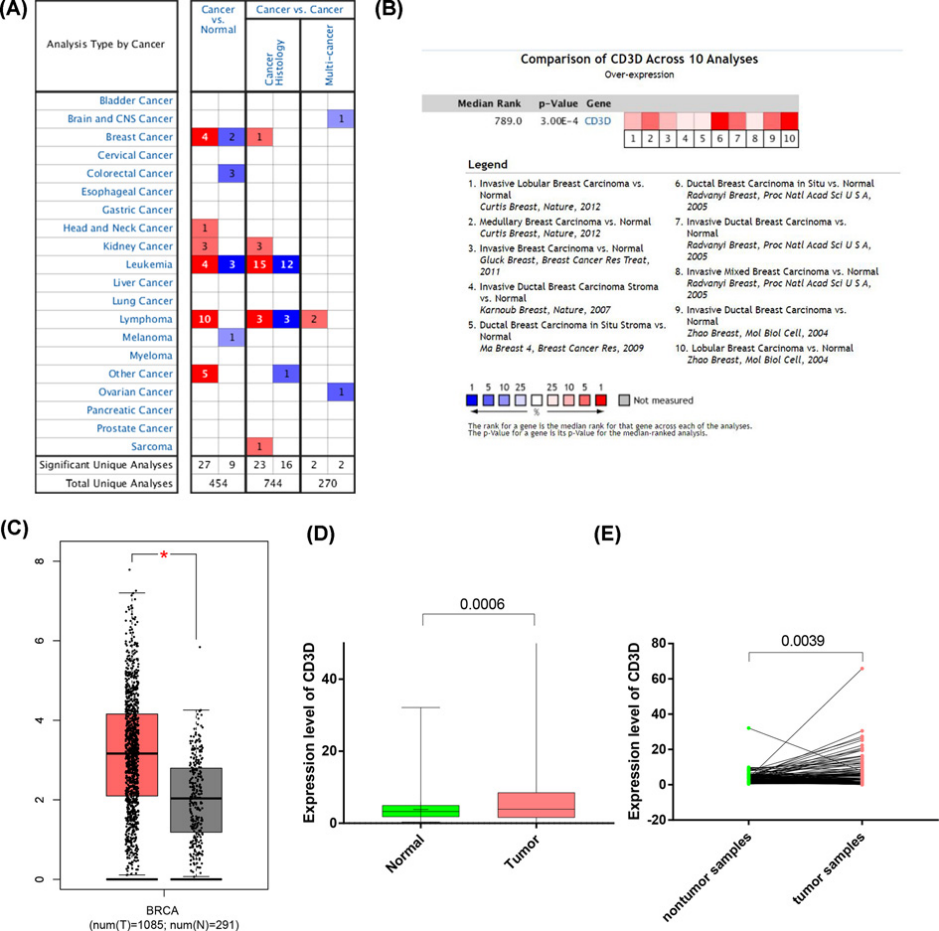
The differentiated CD3D expression between normal and tumor sample (**A**) Overiew of CD3D transcriptional levels in ONCOMINE database. (**B**) Meta-analysis of CD3D based on 10 studies in ONCOMINE database. (**C**) CD3D expression level between normal and tumor sample in GEPIA database. (**D**) CD3D expression level between normal and tumor sample based on TCGA gene expression data. (**E**) CD3D expression level between tumor tissues and paired tissues,* indicates that the mRNA expression between normal and tumor tissue is significantly different with *P*<0.05.

We explored the transcriptional levels of CD3D in BRCA and normal tissues in the ONCOMINE database. The results showed that the transcriptional levels of CD3D in BRCA tissues were significantly elevated (Figure 4A). A meta-analysis based on 10 studies in the ONCOMINE database revealed a significant difference between BRCA and normal tissues (Figure 4B, *P* = 3E-4). Analysis of the GEPIA also indicated that CD3D expression levels were higher in tumor tissues than in normal tissues (Figure 4C). By mining TCGA gene expression data, the mRNA expression of CD3D in tumor samples was significantly higher than that in normal samples (Figure 4D). Pairing analysis showed similar results (Figure 4E).

**Figure 5:**
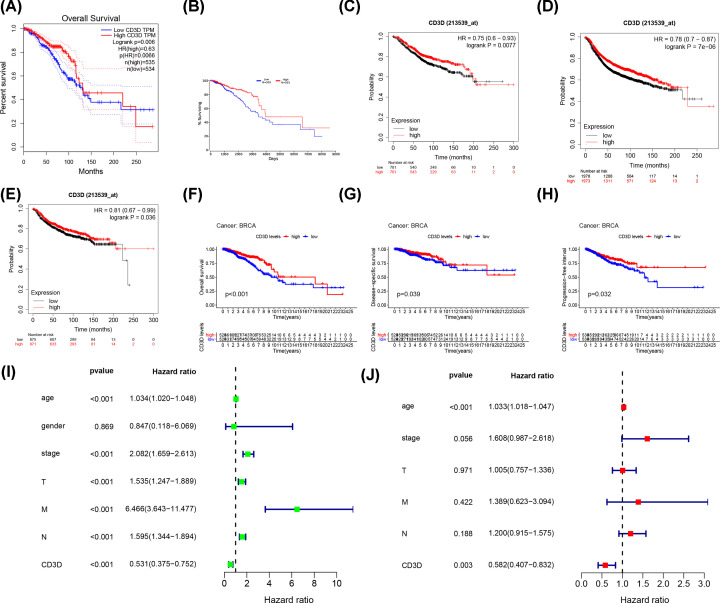
Kaplan–Meier survival analysis, Univariate Cox regression analysis and Multivariate Cox regression analysis of CD3D in patients with BRCA (**A**) Kaplan–Meier survival analysis of OS based on GEPIA. (**B**) Kaplan–Meier survival analysis of OS based on Oncolnc. (**C**) Kaplan–Meier survival analysis of OS based on Kaplan Meier plotter. (**D**) Kaplan–Meier survival analysis of RFS based on Kaplan Meier plotter. (**E**) Kaplan–Meier survival analysis of DMFS based on Kaplan–Meier plotter. (**F**) Kaplan–Meier survival analysis of OS based on TCGA database. (**G**) Kaplan–Meier survival analysis of DSS based on TCGA database. (**H**) Kaplan–Meier survival analysis of PFI based on TCGA database. (**I**) Univariate Cox regression analysis of CD3D based on TCGA database. (**J**) Multivariate Cox regression analysis of CD3D based on TCGA database.

Low CD3D expression was associated with poorer OS in the Online website GEPIA (Logrank *P*-value = 0.006, Figure 5A), Oncolnc (Logrank *P*-value = 0.000598, Figure 5B), Kaplan–Meier plotter indicated that low CD3D expression significantly contributed to worse OS (log-rank *P*=0.0077, Figure 5C), RFS (log-rank *P* = 7E-6, Figure 5D), and DMFS (log-rank *P*=0.036, Figure 5E). BCRA patients with lower CD3D expression were associated with poorer OS (Figure 5F, log-rank *P*<0.001), DSS (log-rank *P*=0.039, Figure 5G), and PFI (log-rank *P*=0.032, Figure 5H). Subsequent univariate COX regression analyses revealed a correlation between OS and age, gender, T, N, M, and stages, as well as CD3D expression (Figure 5I). Multivariate COX regression analysis showed that CD3D expression was an independent prognostic predictor (Figure 5J, *P*=0.003, HR = 0.583, 95% CI = 0.407–0.832). We also evaluated the correlation between differentially expressed CD3D and clinical factors in TIMER (https://cistrome.shinyapps.io/timer/). The COX proportional hazard model indicated that CD3D was significantly associated with the clinical outcome of BRCA patients (*P*=0.030, HR = 0.864, 95% CI = 0.757–0.986, Table 1).

**Table 1:** The cox proportional hazard model of CD3D and clinical factors in BRCA (TIMER)

Factor	coef	HR	95%CI_l	95%CI_H	*P*-value	Sig
Age	0.031	1.032	1.017	1.047	0.000	***
Gender	-0.023	0.977	0.135	7.049	0.982	
raceBlack	-0.175	0.839	0.249	2.824	0.777	
raceWhite	-0.449	0.638	0.198	2.052	0.451	
stage2	0.47	1.6	0.883	2.9	0.121	
stage3	1.271	3.565	1.928	6.594	0.000	***
stage4	2.537	12.642	5.885	27.159	0.000	***
Purity	-0.07	0.933	0.364	2.388	0.885	
CD3D	-0.146	0.864	0.757	0.986	0.030	*

*P<0.05, ***P<0.001.

### CD3D expression correlates with immune-related activities and metabolic pathways

**Figure 6:**
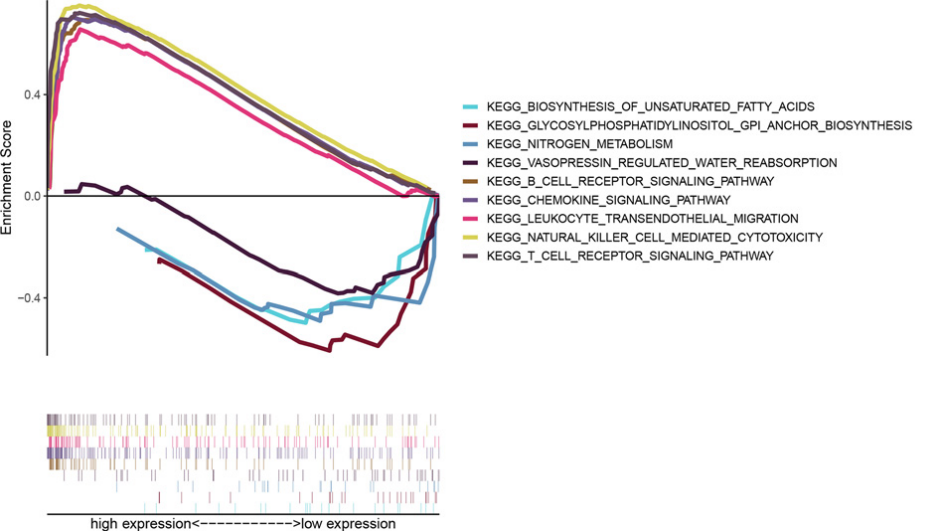
GSEA for samples with high CD3D expression and low expression

Given that CD3D was associated with survival, it could be considered as an independent prognostic predictor, BRCA patients were divided into high- and low- CD3D expression groups according to the median CD3D expression level. We conducted GSEA according to CD3D expression levels. As shown in Figure 6, genes in the high-expression CD3D group were mainly involved in immune-related activities, including KEGG_T_CELL_RECEPTOR_SIGNALING_PATHWAY, KEGG_NATURAL_KILLER_CELL_MEDIATED_CYTOTOXICITY,KEGG_LEUKOCYTE_TRANSENDOTHELIAL_MIGRATION, KEGG_CHEMOKINE_SIGNALING_PATHWAY, KEGG_B_CELL_RECEPTOR_SIGNALING_PATHWAY. While genes in the low-expression CD3D group were enriched in metabolic pathways, including KEGG_VASOPRESSIN_REGULATED_WATER_REABSORPTION, KEGG_NITROGEN_METABOLISM,KEGG_GLYCOSYLPHOSPHATIDYLINOSITOL_GPI_ANCHOR_BIOSYNTHESIS, KEGG_BIOSYNTHESIS_OF_UNSATURATED_FATTY_ACIDS.

### The strong correlation of CD3D expression with T immune cells and immune checkpoints in BRCA

**Figure 7:**
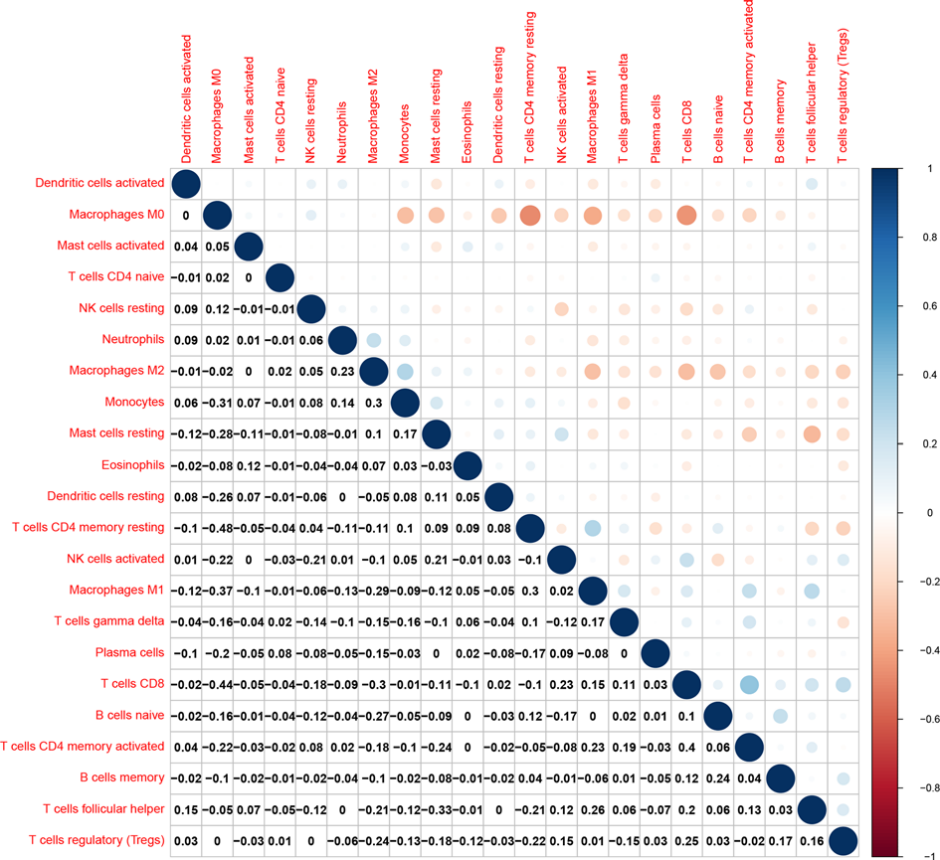
Heatmap to demonstrate the correlation between 21 kinds immune infiltrating lymphocytes

We employed CIBERSORT algorithm to further identify tumor infiltrating immune subsets proportions in BRCA (Supplementary Figure S1). Correlation heatmap revealed the correlation of proportions of different tumor infiltrating immune subpopulations in BRCA (Figure 7). Macrophages M0 had a significant positive correlation with Monocytes, Mast cells resting, T cells CD4 memory resting, Macrophages M1 and T cells CD8. T cells CD4 memory activated and T cells CD8 were most negatively relevant in BRCA.

By mining TIMER, we found that CD3D was an independent prognostic predictor when the confounding factors B_cell, CD8_T cell, CD4_T cell, Macrophage, Neutrophil, Dendritic (Table 2) were corrected.

**Table 2:** The cox proportional hazard model of CD3D and six tumor-infiltrating immune cells in BRCA (TIMER)

Factor	coef	HR	95%CI_l	95%CI_H	*P*-value	Sig
B_cell	-1.182	0.307	0.005	17.223	0.565	
CD8_Tcell	0.121	1.129	0.105	12.076	0.92	
CD4_Tcell	1.355	3.877	0.09	167.414	0.481	
Macrophage	1.422	4.147	0.32	53.686	0.276	
Neutrophil	1.245	3.472	0.027	453.707	0.617	
Dendritic	-0.325	0.722	0.103	5.046	0.743	
CD3D	-0.202	0.817	0.675	0.99	0.039	*

*P<0.05.

**Figure 8:**
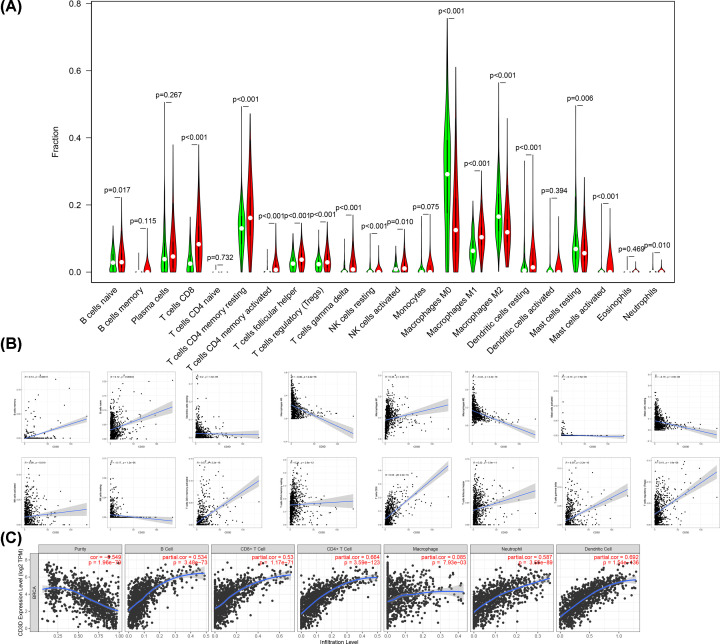
CD3D may affect immune infiltration levels in patients with BRCA (**A**) The Violin plot showed the difference of TIICs fractions between high and low CD3D expression. (**B**) Scatter plot showed CD3D expression correlated 16 kinds of TICs proportion. (**C**) The scatterplots showed CD3D expression correlated immune infiltration cells.

Several analyses were performed to further explore the function of CD3D in TME. The results from the difference analysis showed that B cells naive, T cells CD8, T cells CD4 memory resting, T cells CD4 memory activated, T cells follicular helper, T cells regulatory (Tregs), T cells gamma delta, NK cells resting, NK cells activated, Macrophages M0, Macrophages M1, Macrophages M2, Dendritic cells resting, Mast cells resting, Mast cells activated and Neutrophils were affected by CD3D expression. And B cells naïve, T cells (T cells CD8, T cells CD4 memory resting, T cells CD4 memory resting, T cells CD4 memory activated, T cells follicular helper, T cells regulatory (Tregs), T cells gamma delta), NK cells (NK cells resting, NK cells activated), Macrophages M1, Dendritic cells resting, Mast cells activated and Neutrophils were at higher proportions in the high CD3D expression group. Macrophages M0, Macrophages M2 and Mast cells resting were significantly downregulated in the high CD3D group (Figure 8A). Next, we conducted a correlation analysis between CD3D and immune cells, with *P*-value <0.05 and Spearman Correlation Coefficient *r*>0.5. The results demonstrated that CD3D had the strongest correlation with CD8 + T cells and T cells CD4 memory activated (Figure 8B). For further validation, we investigated the association between CD3D and TIICs in TIMER. CD3D was positively associated with B cell, CD8+T cell, CD4+T cell, Macrophage, Neutrophil, and Dendritic Cell. TIMER also revealed that CD3D was significantly associated with CD8 + T and CD4+ T cells (Figure 8C). The above difference analysis, correlation analysis and TIMER indicated that CD3D had a strong correlation with T cells, particularly with CD8 + T cells.

In addition, we performed correlation analysis between CD3D and infiltrating lymphocytes, we performed an analysis between CD3D expression and the markers of infiltrating lymphocytes. This analysis revealed that CD3D expression had a strong positive correlation with CD8 + T cells, T cell exhaustion, T cell (general), CD4 + T cells, Tfh, Th1, Treg (Table 3).

**Table 3:** Spearman correlation analysis between CD3D and infiltrating lymphocytes in patients with breast carcinoma

Terms	Markers	*R*	*P* (two-tailed)	*P* value summary
CD4 + T cells	CXCR4	0.5195	<0.0001	****
	CD40LG	0.843	<0.0001	****
	CD4	0.7026	<0.0001	****
CD8 + T cells	CD8A	0.8358	<0.0001	****
	CD8B	0.7073	<0.0001	****
T cell (general)	CD2	0.9395	<0.0001	****
	CD3G	0.8596	<0.0001	****
	CD3E	0.9579	<0.0001	****
	CD28	0.7888	<0.0001	****
T cell exhaustion	PDCD1LG2	0.52	<0.0001	****
	CD244	0.7031	<0.0001	****
	PDCD1	0.8546	<0.0001	****
	GZMB	0.6458	<0.0001	****
	CD274	0.4508	<0.0001	****
	BTLA	0.7791	<0.0001	****
	CD96	0.8383	<0.0001	****
	TIGIT	0.8867	<0.0001	****
	TGFBR1	-0.04661	0.1208	ns
	CTLA4	0.81	<0.0001	****
	HAVCR2	0.4212	<0.0001	****
	KDR	0.02114	0.4818	ns
	IDO1	0.2598	<0.0001	****
Tfh	IL21	0.6302	<0.0001	****
Th1	STAT1	0.3859	<0.0001	****
	TBX21	0.8889	<0.0001	****
	IFNG	0.5598	<0.0001	****
	STAT4	0.8252	<0.0001	****
Th2	STAT6	0.1493	<0.0001	****
	STAT5A	0.3054	<0.0001	****
Treg	STAT5B	0.09421	0.0017	**
	IL2RA	0.3665	<0.0001	****
Dendritic cell	NRP1	0.01807	0.5478	ns
	HLA-DRA	0.6879	<0.0001	****
	CD1C	0.5852	<0.0001	****
	HLA-DPA1	0.6452	<0.0001	****
	ITGAX	0.476	<0.0001	****
Macrophage	CD163	0.2508	<0.0001	****
Monocyte	CSF1R	0.3285	<0.0001	****
Natural killer cell	NCAM1	-0.04226	0.1596	ns
	FCGR3A	0.2764	<0.0001	****
B cell	CD79A	0.5748	<0.0001	****

^**P<0.01, ****P<0.0001.^

**Figure 9:**
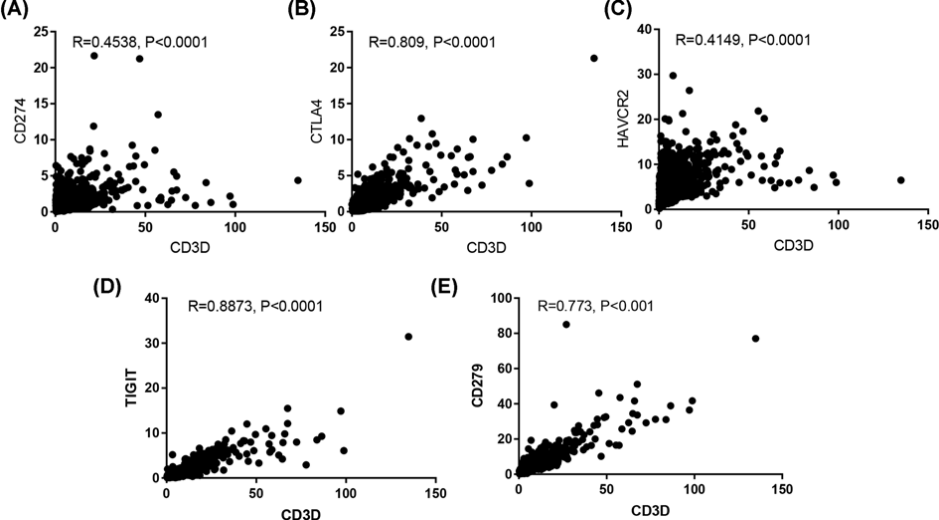
CD3D is positively correlated with PD-L1 (CD274), CTLA-4, TIGIT, TIM-3 (HAVCR2) and PD-1 (CD279)

The above analysis indicated that CD3D is strongly associated with T cells. It was known that ICB therapy presents promising clinical effects. Next, we performed a correlation analysis between CD3D and immune checkpoints and found that CD3D positively correlated with immune checkpoints, including PD-L1 (CD274), TIGIT, CTLA-4, TIM-3 (HAVCR2) and PD-1 (CD279) (Figure 9).

### CD3D was highly expressed in CD8 + T cells based on Single cell RNA-seq analysis

**Figure 10:**
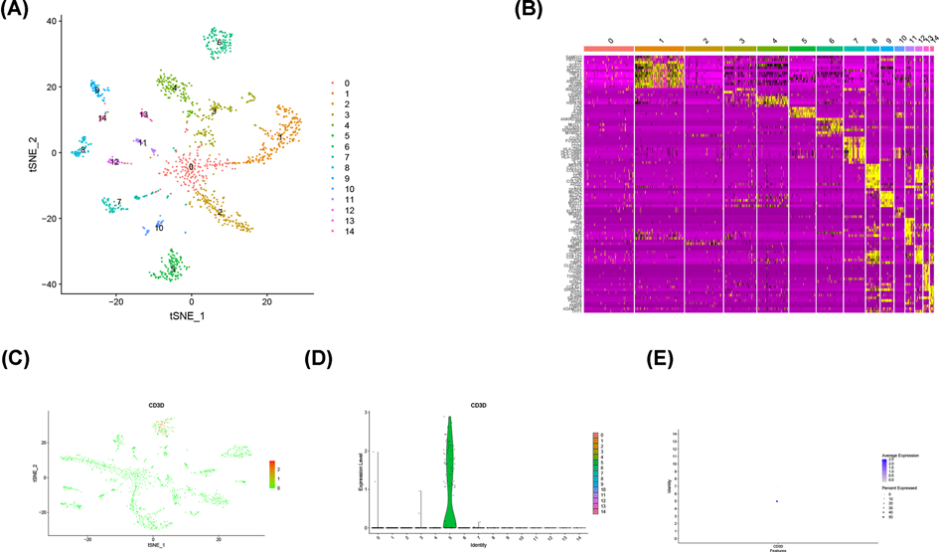
CD3D was highly expressed in CD8 + T cells based on Single cell RNA-seq data analysis (**A**) t-SNE representation of 14 subgroups. (**B**) Heatmap showing expression of marker genes potentially associated clusters. (**C**) Expression levels of CD3D in clusters are plotted onto the t-SNE map. (**D**) Violin plots showing the expression level of CD3D across the clusters. (**E**) Bubble diagram showing the expression level of CD3D across the clusters. 0: Epithelial cells, 1: Epithelial cells, 2: Chondrocytes, 3: Epithelial cells, 4: Epithelial cells, 5: CD8+T-cells, 6: Epithelial cells, 7: Macrophages, 8: Fibroblasts, 9: Chondrocytes, 10: B-cells,11: Chondrocytes, 12: Chondrocytes, 13: Endothelial cells, 14: Fibroblasts.

Principle component analysis was applied to explore the cellular composition based on variably expressed genes across all cells, 15 clusters were identified (Figure 10A) and the heatmap demonstrated the cluster-specific marker genes in the 15 clusters (Figure 10B). Expression level of CD3D in each clusters are plotted onto t-SNE map (Figure 10C), Violin plots (Figure 10D) and bubble diagram (Figure 10E), which indicated that CD3D was highly expressed in CD8 + T cells.

## Discussion

TME is a complex molecular and cellular network that consists of various cell types and molecules. The main cellular components of the TME are stromal cells and recruited immune cells [17]. Increasing evidence indicates that TME plays a crucial role in the growth and progression of cancer. We analyzed data involving BRCA in the TCGA dataset and demonstrated that immune components in TME significantly correlated with OS. Infiltration of immune cells, particularly tumor TILs, have been reported to correlate with survival in multiple types of cancer, and TILs account for 70–80% of the immune cells in BRCA. Therefore, it is crucial to develop an effective immunotherapy for BRCA. Even so, Even so, Immunotherapy for BRCA is still considered immunologically quiescent [18], Recent articles have reported that increased TILs are associated with a higher pathologic response to neoadjuvant therapy [19–21]. As a vital portion of immunotherapy, ICB has revolutionized cancer therapy. Although immune checkpoint inhibitors (ICIs) have not been approved for patients with advanced BRCA, several randomized phase 3 ICB monotherapy trials are ongoing and small trials have presented some promising clinical results [2]. Therefore, it is necessary to explore novel biomarkers associated with immune-based therapy in BRCA.

In the present study, we performed a systematic analysis of BRCA in TCGA database, which identified CD3D as a novel biomarker. The studies reveal that tumor samples expressed higher levels of CD3D. However, the low CD3D group exhibited lower percentage of survival than the high CD3D group, which was contrary to what had been expected. We hypothesized that CD3D may be a tumor suppressor gene, which plays an antitumor role in BRCA. To further analyze the significance of CD3D in BRCA, we performed a GSEA analysis, which showed that the high CD3D expression group was associated with immune-related signaling pathways, and the low CD3D expression group was enriched in metabolic pathways. Single cell RNA-seq analysis indicated that CD3D may participate activating CD8 + T cells to suppress BRCA. These results indicate that metabolic-dominant converts to immune-dominant with increased CD3D expression levels, which thus suggests that CD3D could inhibit tumors by activating the immune component in TME.

It is well-known that CD3D participates in the promotion of T-cell activation [13]. However, the significance of CD3D in BRCA is not clear. We applied the CIBERSORT algorithm to analyze the association between CD3D and infiltrating lymphocytes. The difference and correlation analysis showed that CD3D had a strongly positive correlation with TILs in BRCA. It is known that TILs account for 70–80% of immune cells in BRCA, and play a crucial role in tumor immunity. Therefore, the positive correlation between CD3D and TILs suggested that high levels of CD3D expression may increase T cell immune infiltration in TME and induce antitumor immunity by activating TILs in BRCA.

As a notable immunotherapy, ICB has emerged as a powerful tool in the management of multiple cancer types, and some small BRCA trials have shown meaningful clinical results. It is known that PD-L1 and PD-L2 interact with PD-1 to inhibit T cell activation [22]. Also, CTLA-4 could inhibit CD4 + and CD8 + T cells by influencing the function of APC. TIM-3 has a double role in tumor immune response [23]. In the present study, CD3D expression showed a strong positive correlation with immune checkpoints, including PD-L1,PD-L2, CTLA-4 and TIM-3, which suggests a CD3D-mediated regulation of T cell functions in BRCA. Several limitations of the present study should be acknowledged. First, the patient cohorts from TCGA were retrospective, and prospective cohorts are needed to validate our results. Second, further in vivo biological experiments should be performed to unravel the potential mechanisms of CD3D in BRCA.

In conclusion, we identified CD3D as a prognostic biomarker in BRCA. High CD3D expression correlated with high percentage of survival, higher lymphocyte infiltration levels and immune checkpoint expression, which may offer an additional insight into therapeutics of BRCA.

## Supplementary Material

Supplementary Figure S1

## Data Availability

The original contributions presented in the study are publicly available. These data can be found here: (https://portal.gdc.cancer.gov/).

## Abbreviations

APC: antigen-presenting cell
BRCA: breast carcinoma
CTL: cytotoxic T cell
DEG: differential expression gene
GSEA: Gene Set Enrichment Analysis
ICI: immune checkpoint inhibitor
NES: normalized enrichment score
PPI: protein–protein interaction
TCGA: The Cancer Genome Atlas
Tregs: T cells regulatory
