# Supplementary figures and images for: Comprehensive analysis reveals a prognostic and therapeutic biomarker CD3D in the breast carcinoma microenvironment
Source: Biosci Rep. 2021 Jan 7;41(1):BSR20202898. doi: 10.1042/BSR20202898 (PMC7791551; doi:10.1042/BSR20202898)

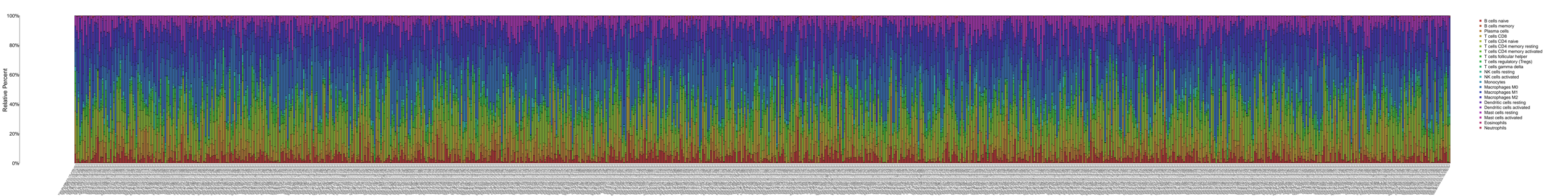

Supplement: Supplementary Figure S1 [file BSR-2020-2898_supp.pdf]
